# Supplementary figures and images for: UPRER–immunity axis acts as physiological food evaluation system that promotes aversion behavior in sensing low-quality food
Source: eLife. 2024 Sep 5;13:RP94181. doi: 10.7554/eLife.94181 (PMC11377039; doi:10.7554/eLife.94181)

Figure 2—source data 2

Figure 2E

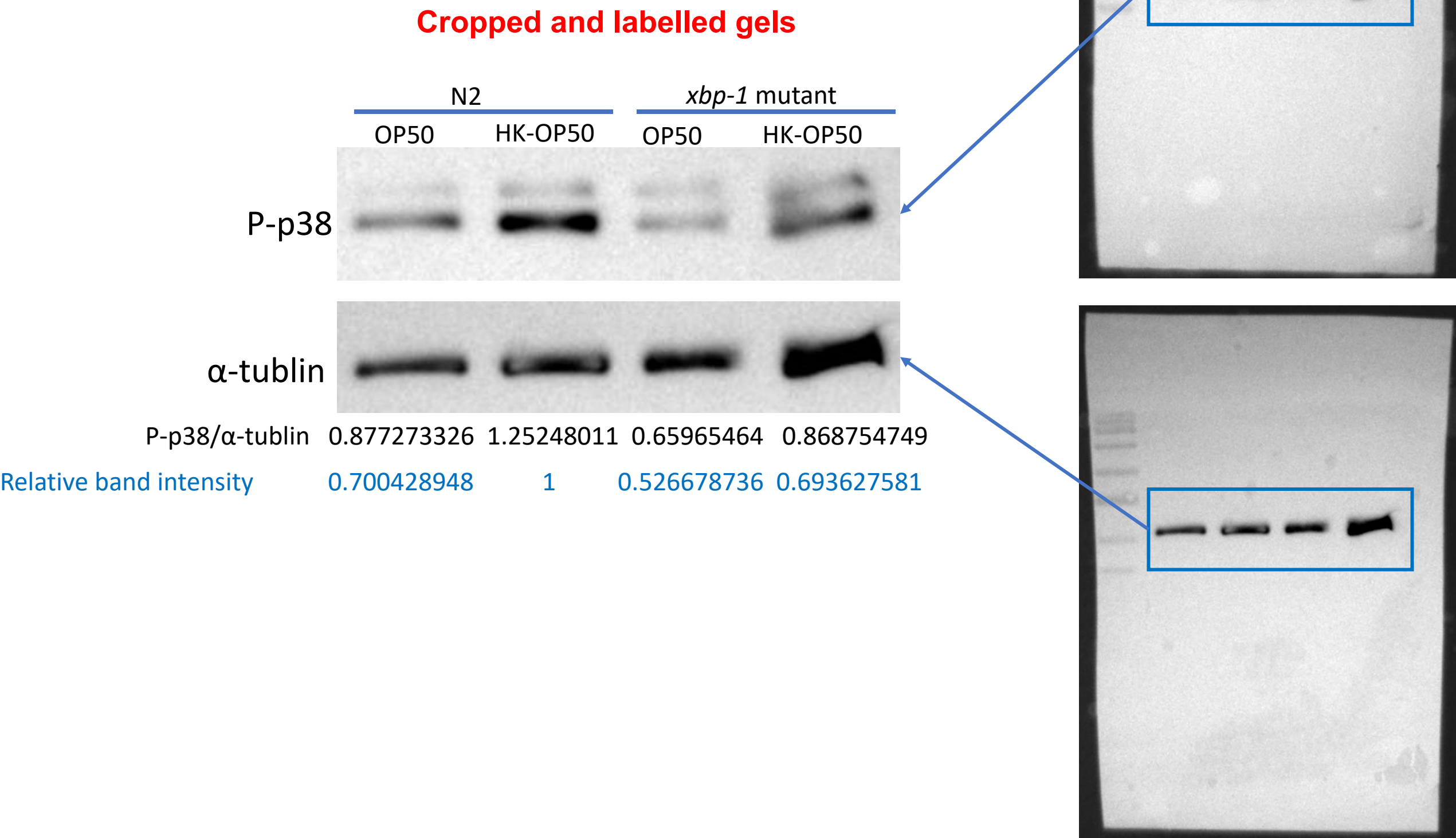

Supplement: Figure 2—source data 2. [file elife-94181-fig2-data2.pdf]

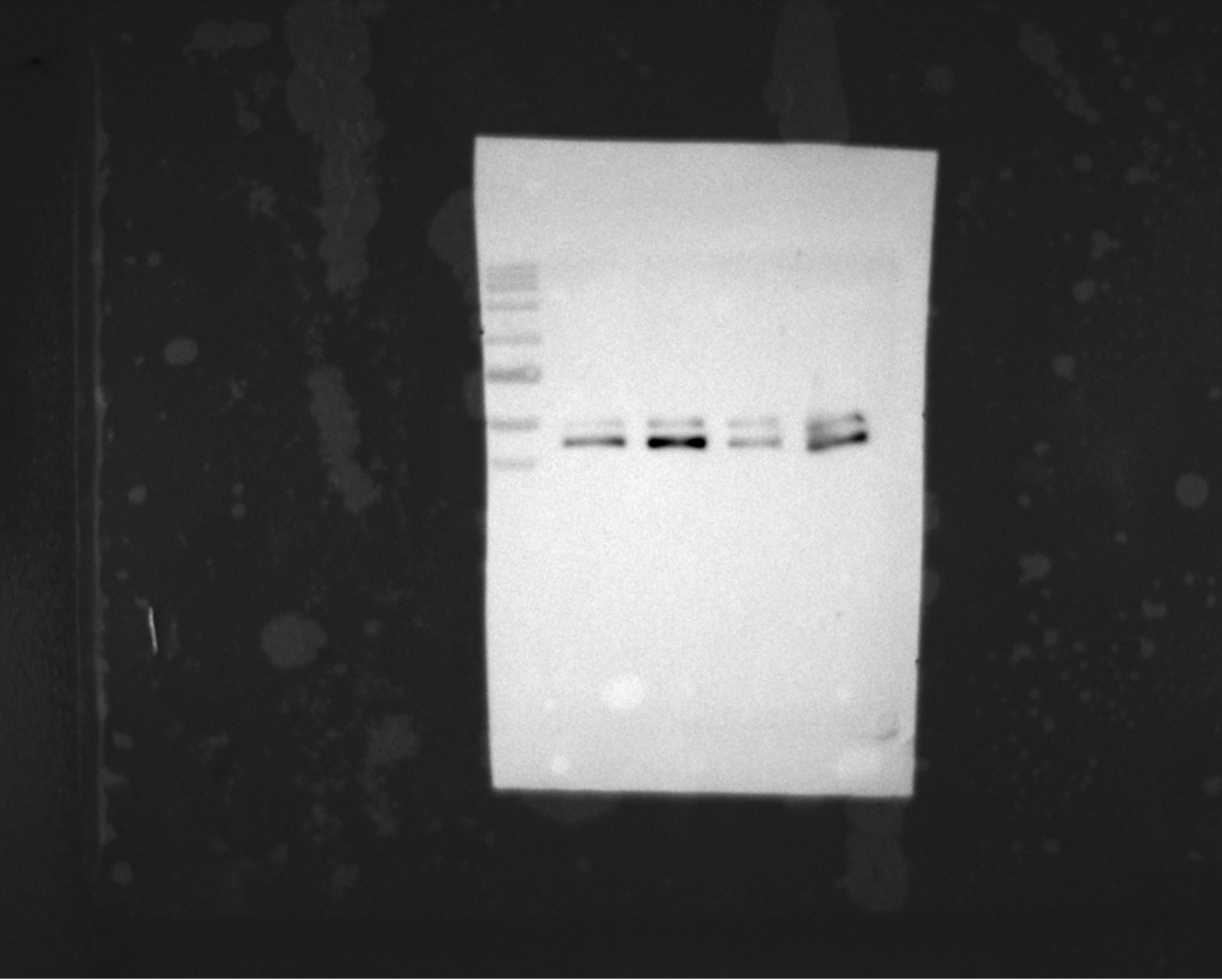

Supplement: Figure 2—source data 3. [file elife-94181-fig2-data3.zip › Figure 2E-1.tif]

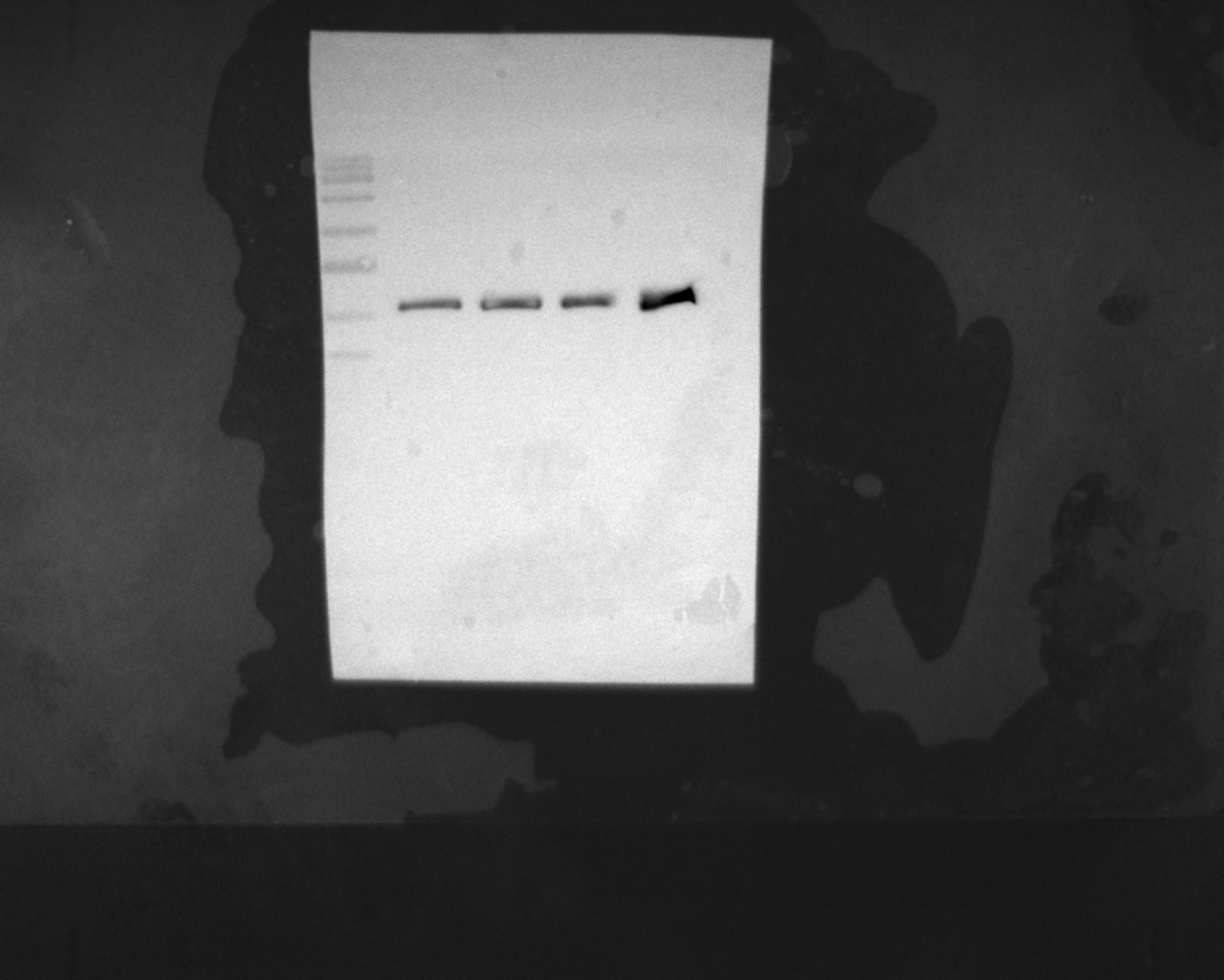

Supplement: Figure 2—source data 3. [file elife-94181-fig2-data3.zip › Figure 2E-2.tif]

Figure 3—source data 2

Figure 3G

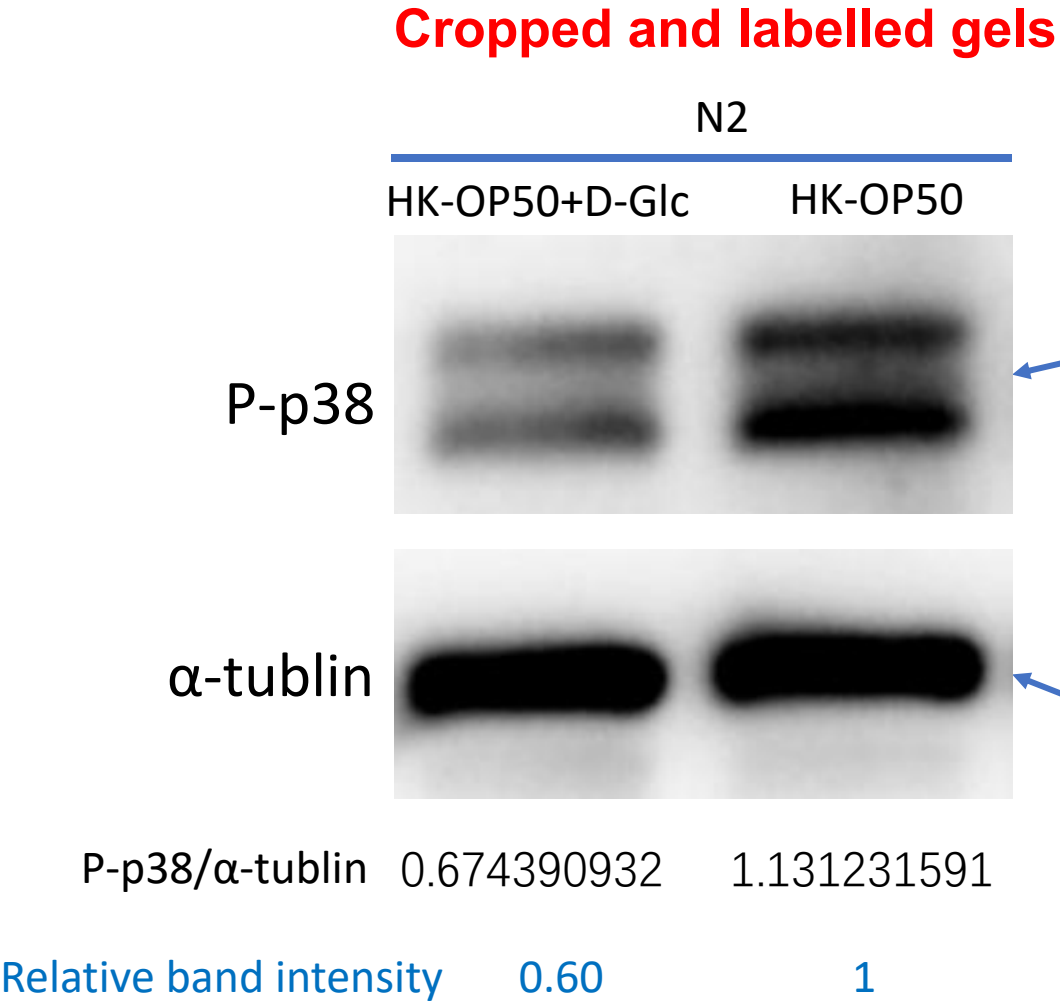

**Raw unedited gels**

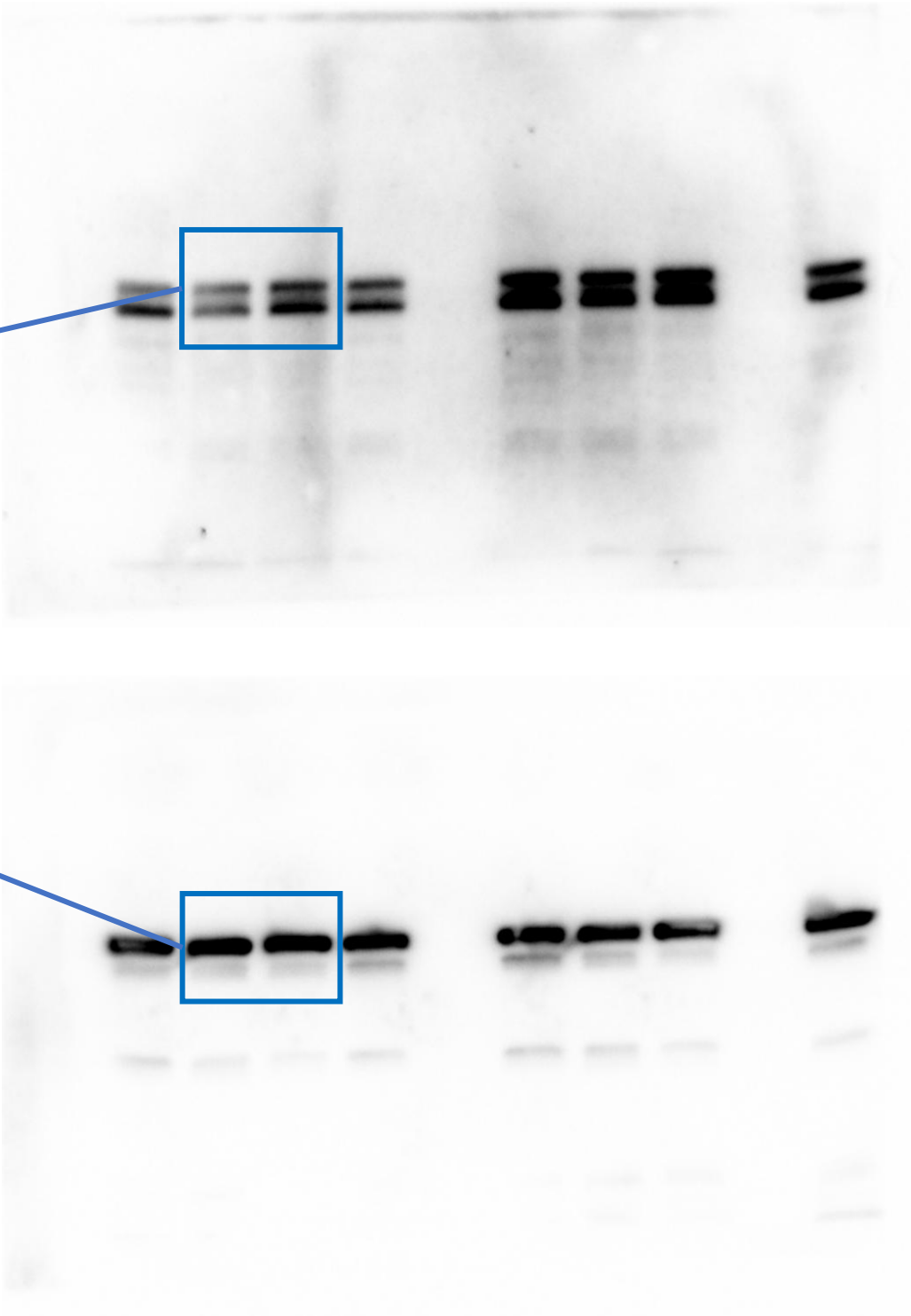

Supplement: Figure 3—source data 2. [file elife-94181-fig3-data2.pdf]

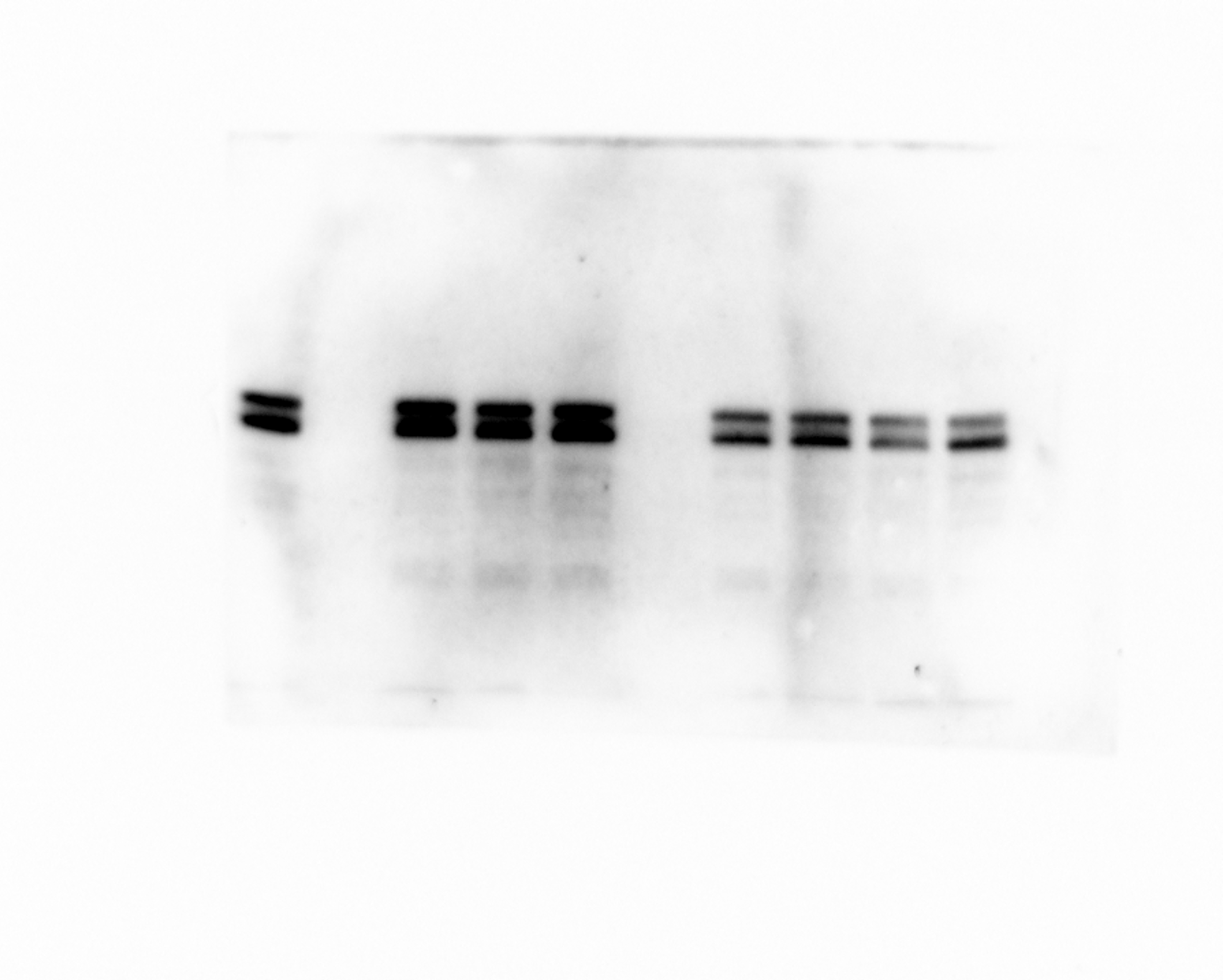

Supplement: Figure 3—source data 3. [file elife-94181-fig3-data3.zip › Figure 3G-1.tif]

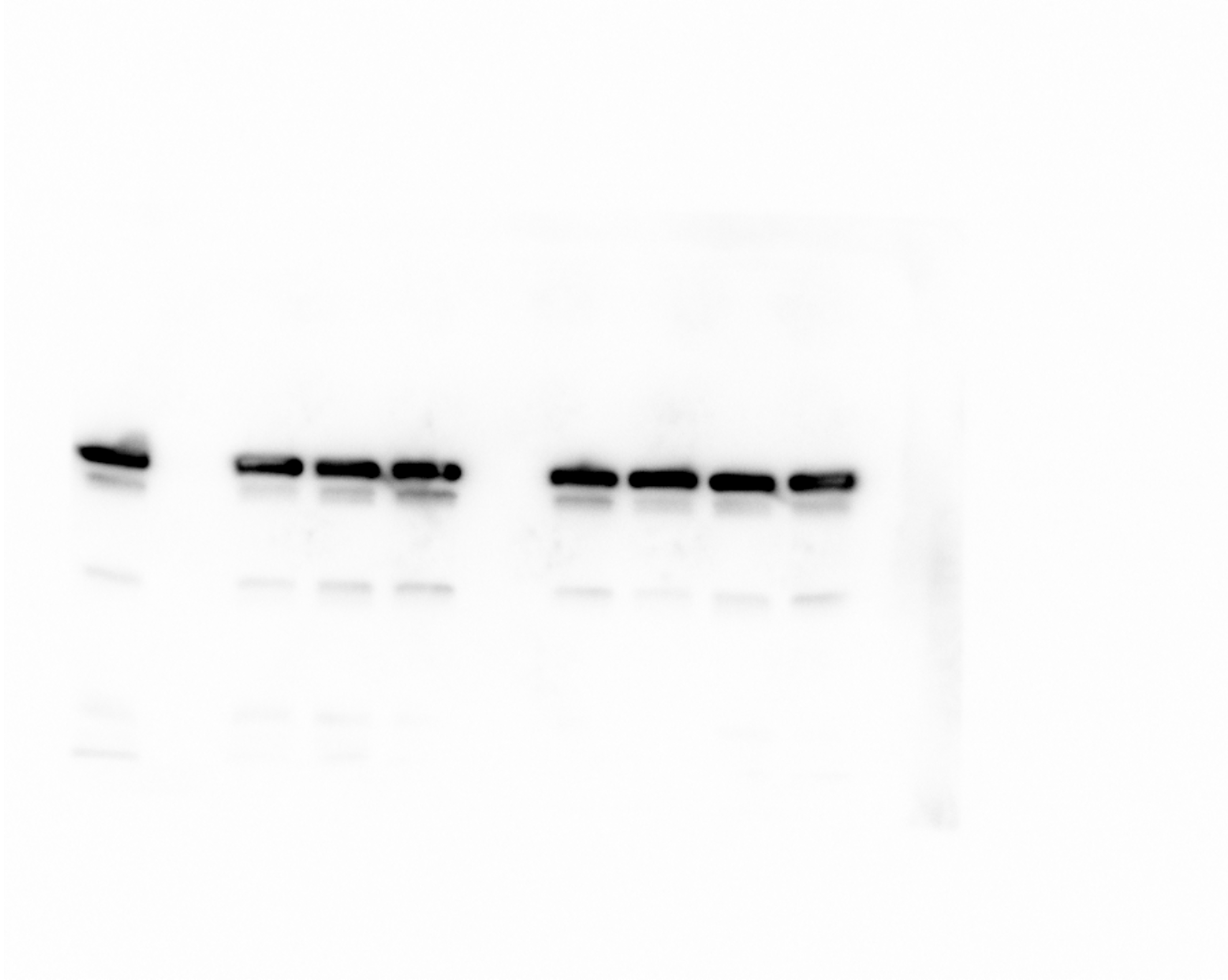

Supplement: Figure 3—source data 3. [file elife-94181-fig3-data3.zip › Figure 3G-2.tif]

Figure 4—source data 2

Figure 4F

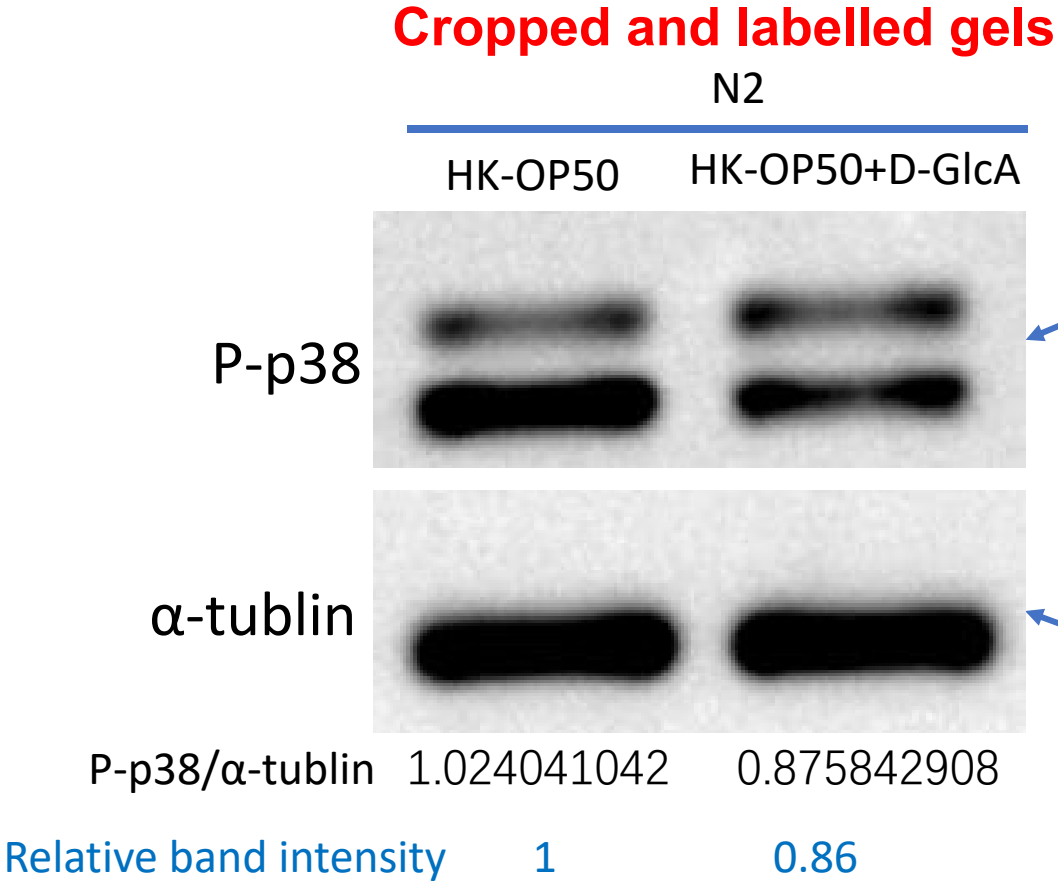

Raw unedited gels

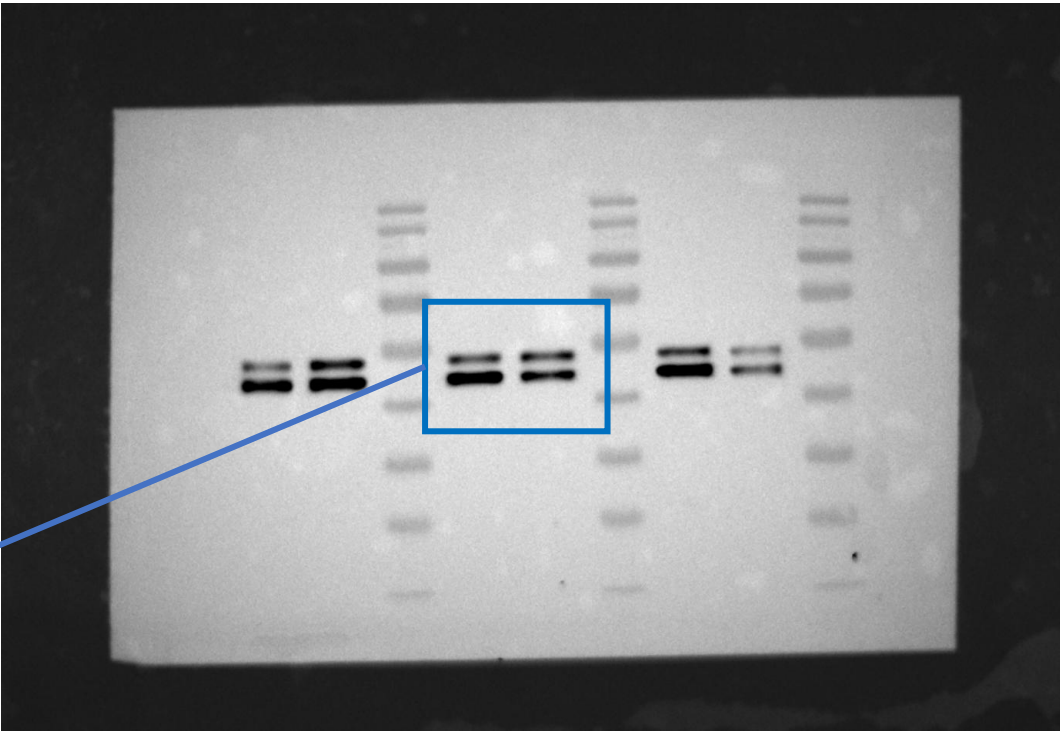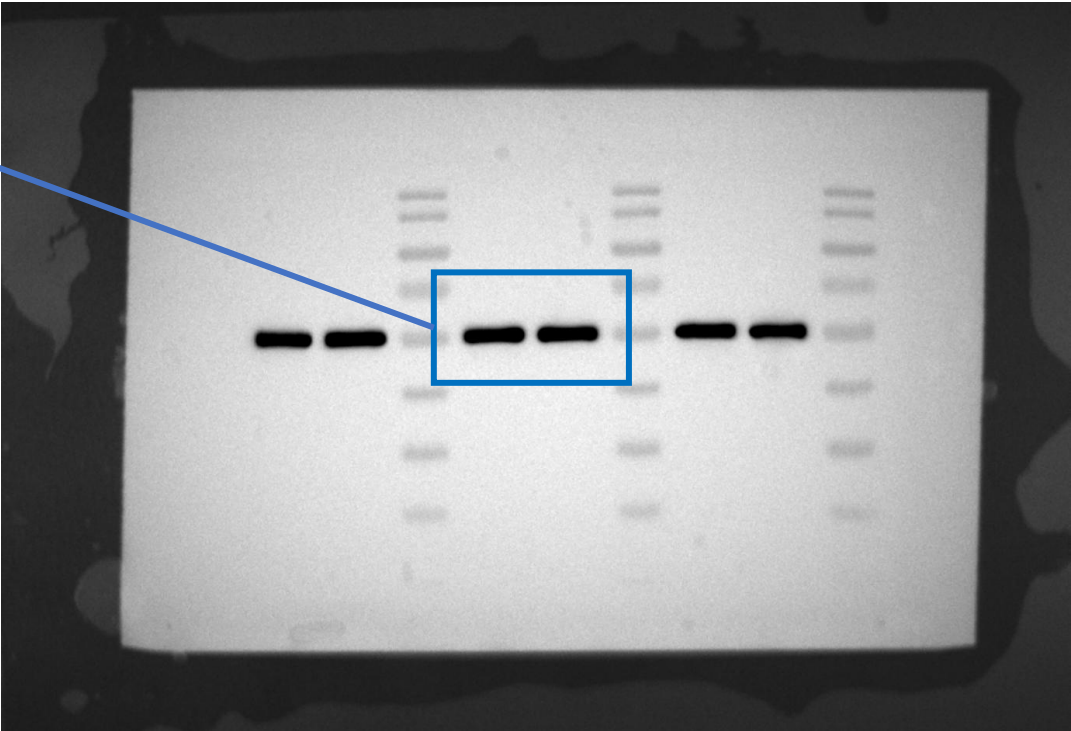

Figure 4—source data 2

Figure 4G

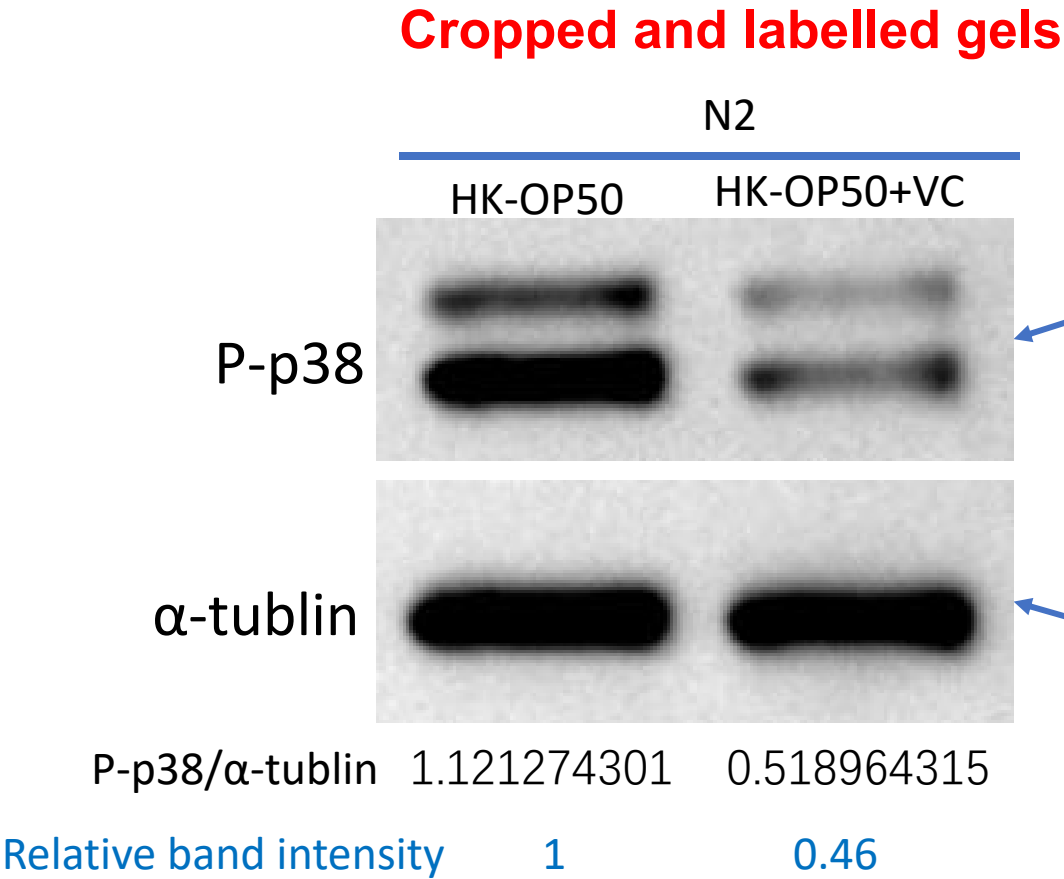

Raw unedited gels

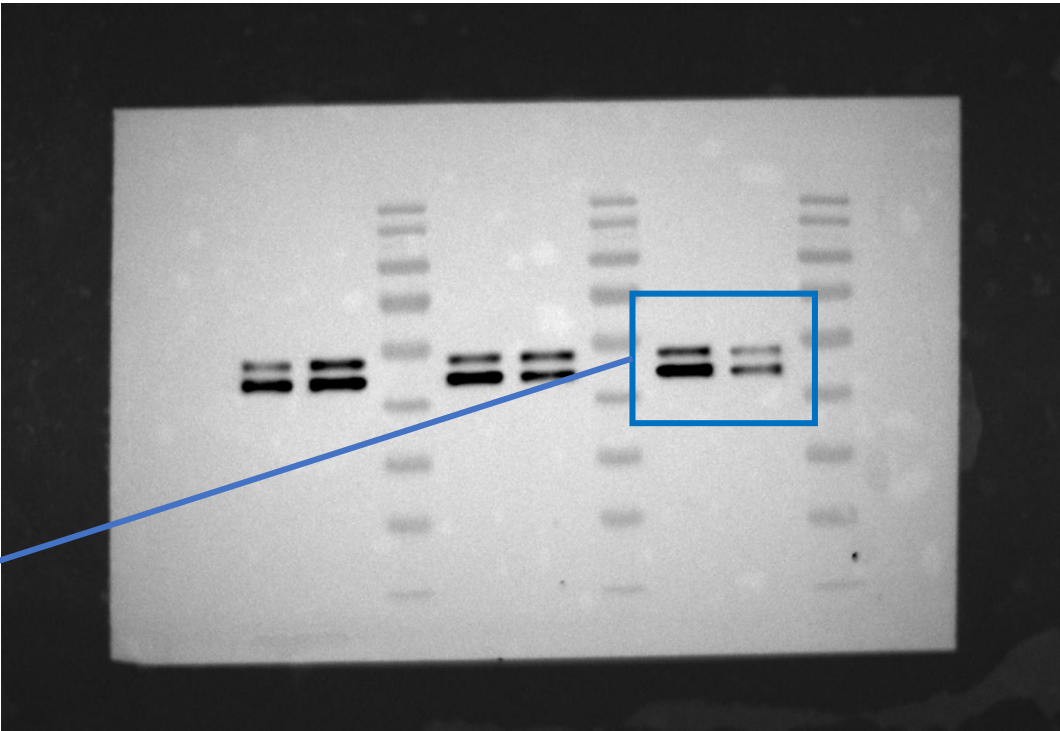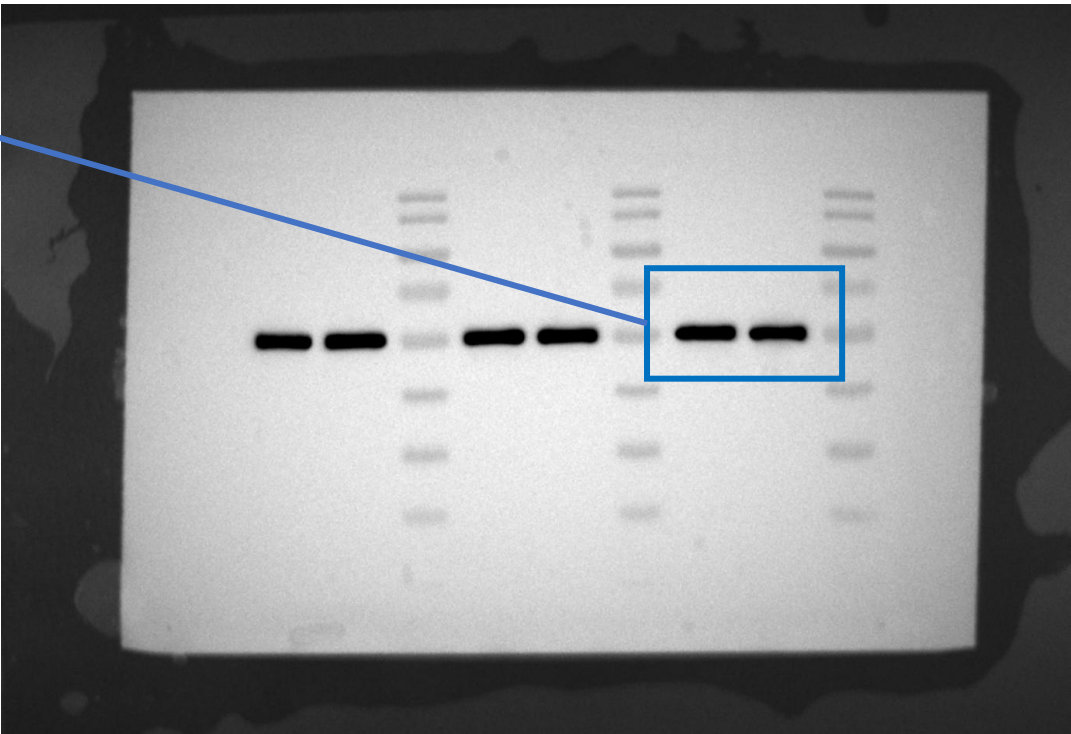

Supplement: Figure 4—source data 2. [file elife-94181-fig4-data2.pdf]

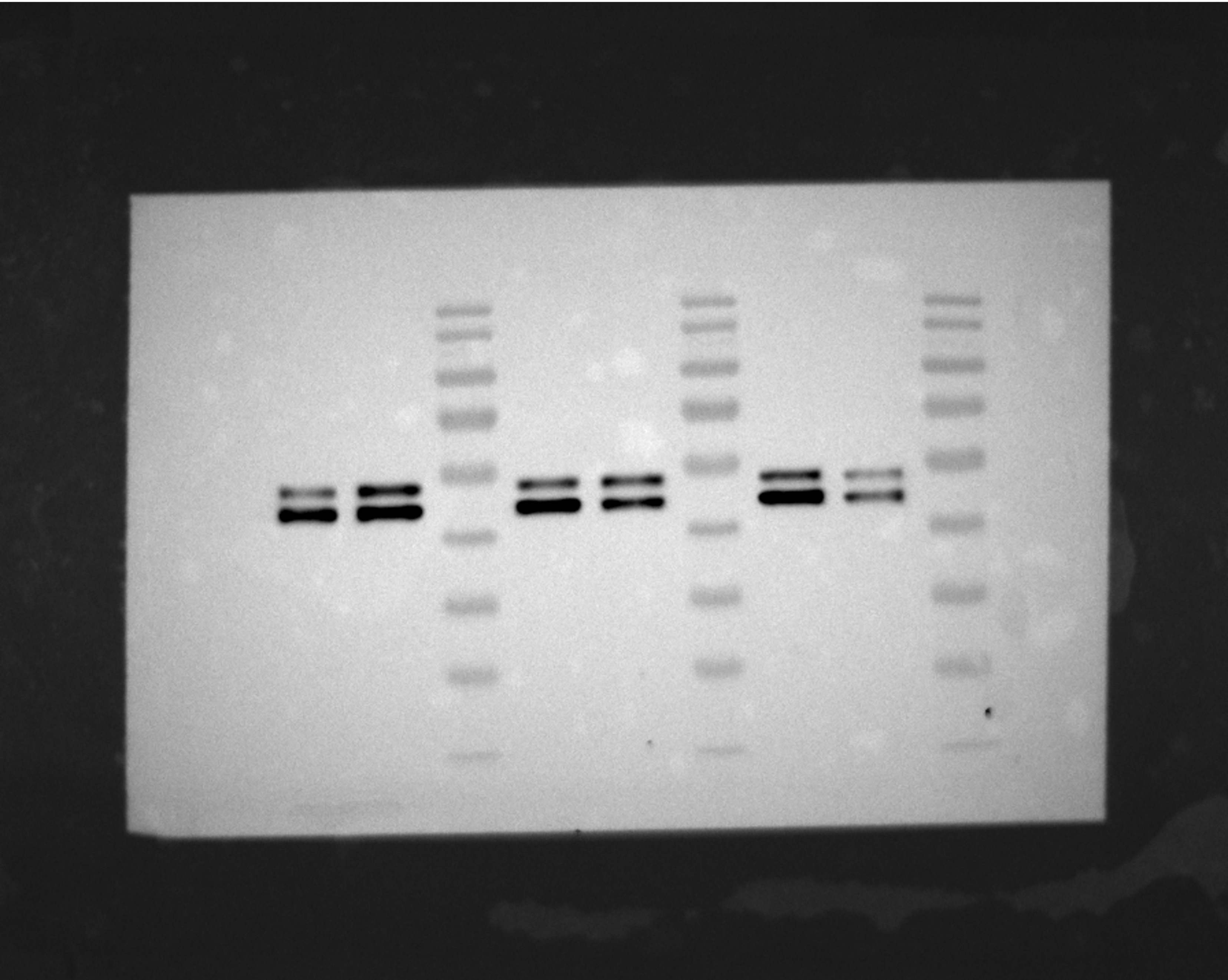

Supplement: Figure 4—source data 3. [file elife-94181-fig4-data3.zip › Figure 4G-F-1.tif]

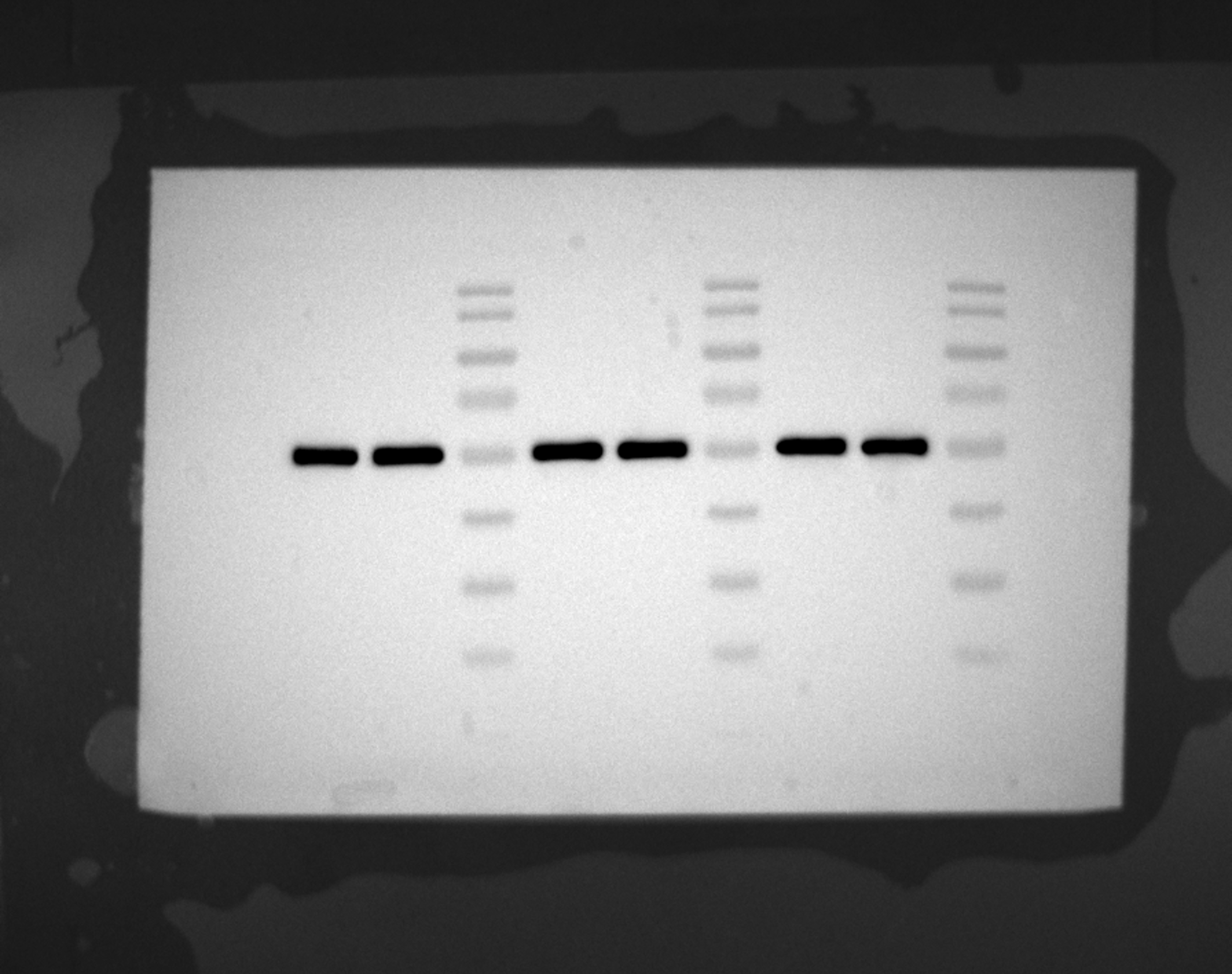

Supplement: Figure 4—source data 3. [file elife-94181-fig4-data3.zip › Figure 4G-F-2.tif]

Figure 5—source data 2

Figure 5-figure supplement 1-D

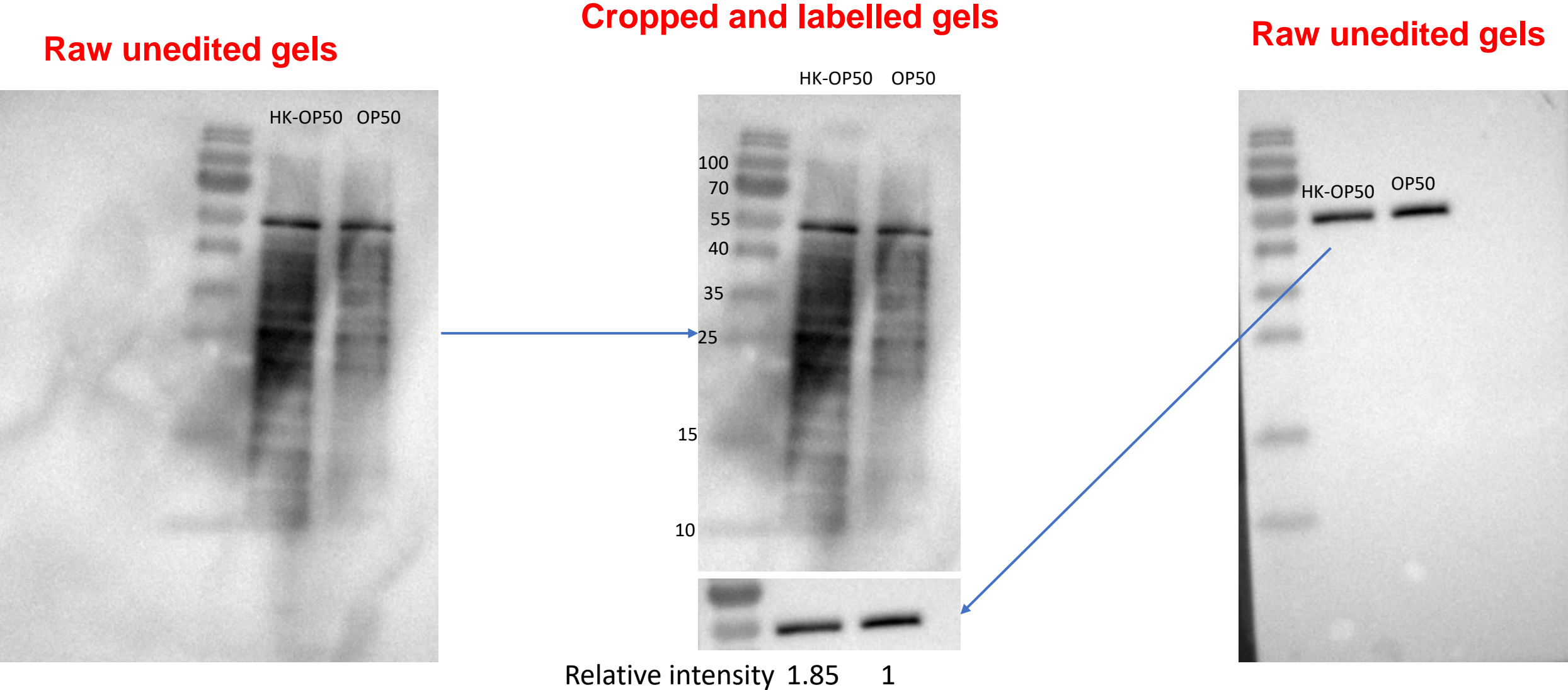

Supplement: Figure 5—figure supplement 1—source data 1. [file elife-94181-fig5-figsupp1-data1.pdf]

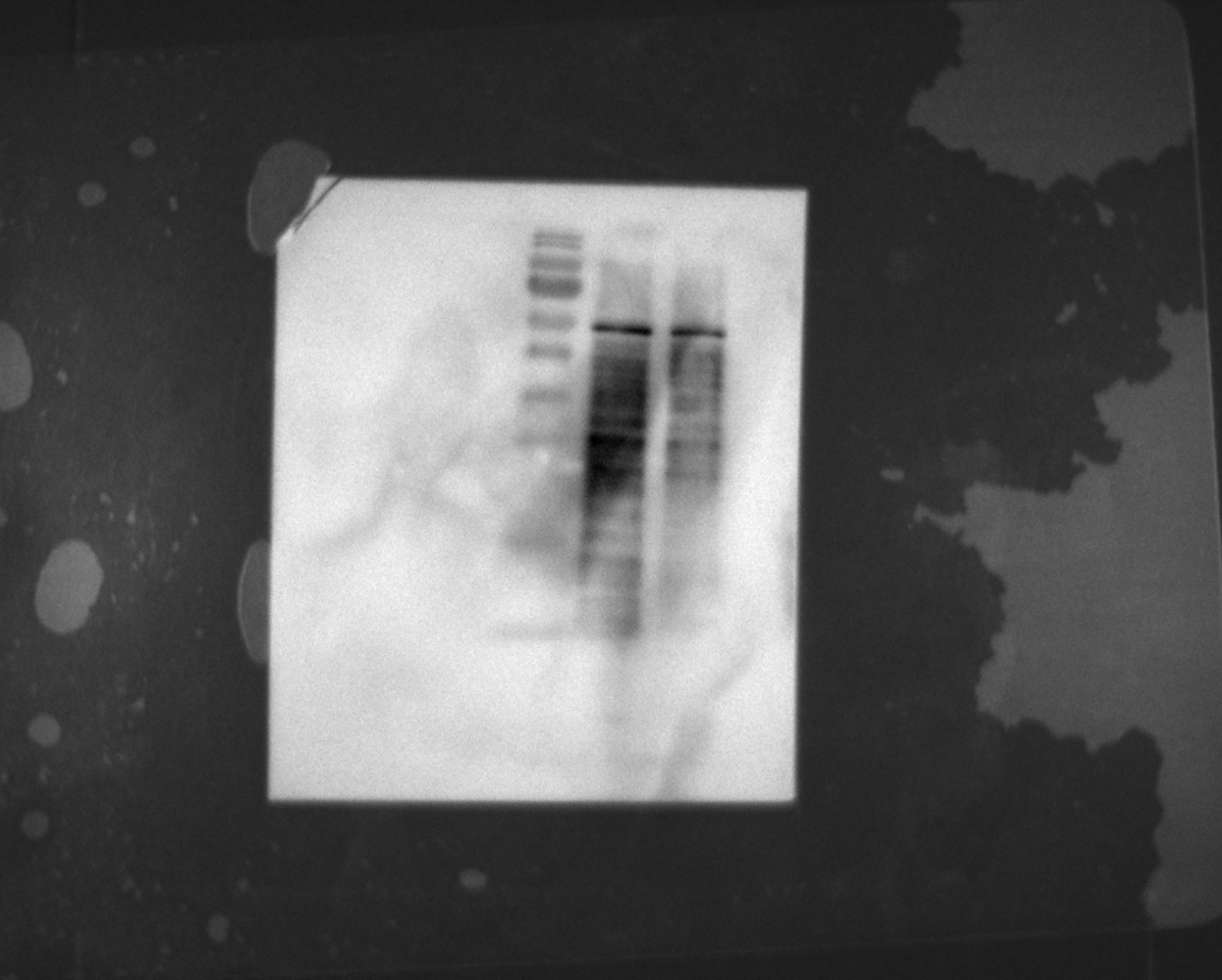

Supplement: Figure 5—figure supplement 1—source data 2. [file elife-94181-fig5-figsupp1-data2.zip › Figure 5-figure supplement 1D-1.tif]

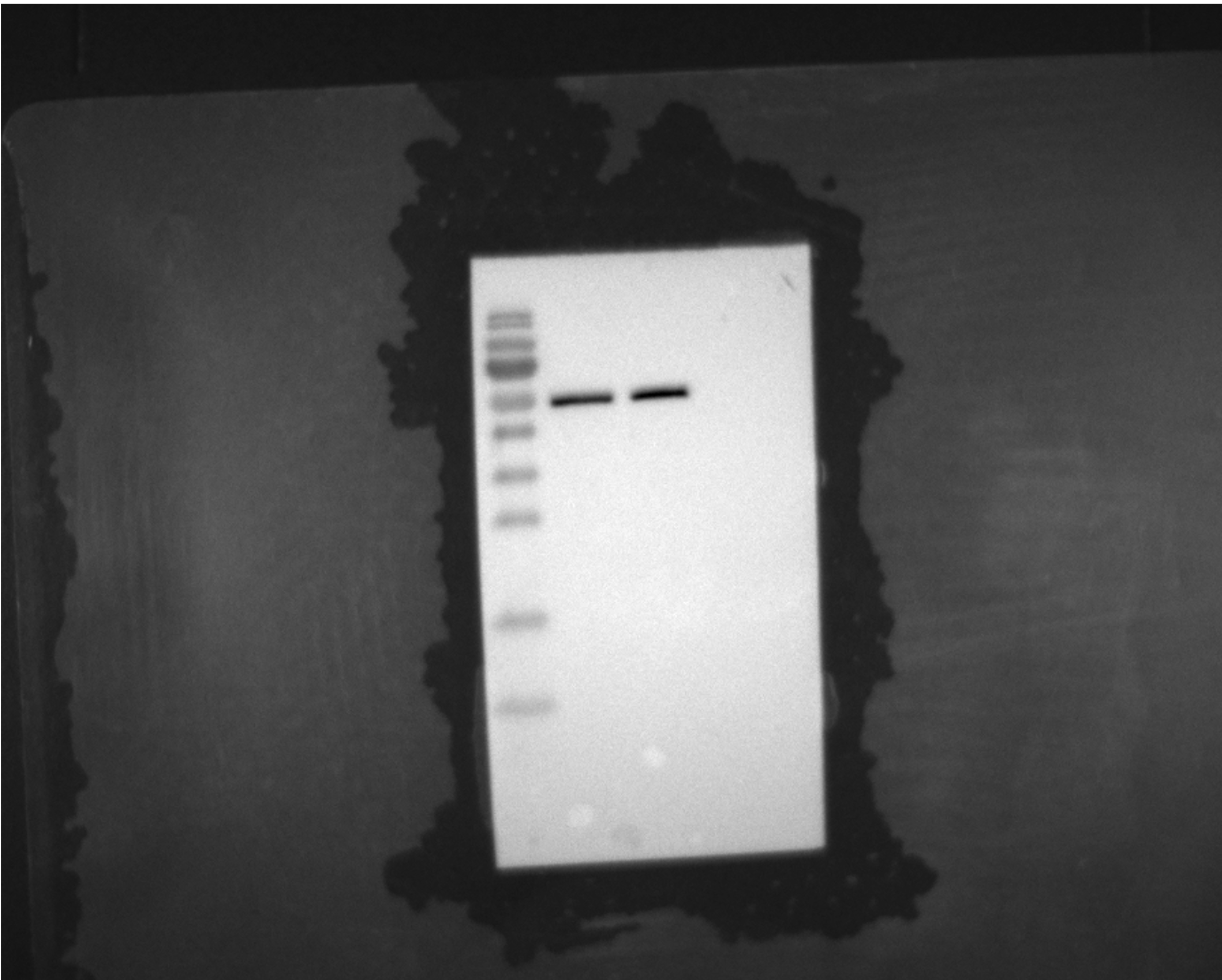

Supplement: Figure 5—figure supplement 1—source data 2. [file elife-94181-fig5-figsupp1-data2.zip › Figure 5-figure supplement 1D-2.tif]
